# Patient recruitment strategies for adaptive enrichment designs with time-to-event endpoints

**DOI:** 10.1186/s12874-019-0800-2

**Published:** 2019-07-22

**Authors:** Ryuji Uozumi, Shinjo Yada, Atsushi Kawaguchi

**Affiliations:** 1 Department of Biomedical Statistics and Bioinformatics, Kyoto University Graduate School of Medicine, 54 Kawahara-cho, Shogoin, Sakyo-ku, Kyoto, 606-8507 Japan; 2 Biostatistics Department I, Data Science Division, A2 Healthcare Corporation, 1-4-12, Utsubohommachi, Nishi-ku, Osaka, 550-0004 Japan; 3 Faculty of Medicine, Saga University, 5-1-1 Nabeshima, Saga, 849-8501 Japan

**Keywords:** Adaptive enrichment designs, Time-to-event endpoints, Patient recruitment, Interim analysis, Targeted therapies

## Abstract

**Background:**

Adaptive enrichment designs for clinical trials have great potential for the development of targeted therapies. They enable researchers to stop the recruitment process for a certain population in mid-course based on an interim analysis. However, adaptive enrichment designs increase the total trial period owing to the stoppage in patient recruitment to make interim decisions. This is a major drawback; it results in delays in the submission of clinical trial reports and the appearance of drugs on the market. Here, we explore three types of patient recruitment strategy for the development of targeted therapies based on the adaptive enrichment design.

**Methods:**

We consider recruitment methods which provide an option to continue recruiting patients from the overall population or only from the biomarker-positive population even during the interim decision period. A simulation study was performed to investigate the operating characteristics by comparing an adaptive enrichment design using the recruitment methods with a non-enriched design.

**Results:**

The number of patients was similar for both recruitment methods. Nevertheless, the adaptive enrichment design was beneficial in settings in which the recruitment period is expected to be longer than the follow-up period. In these cases, the adaptive enrichment design with continued recruitment from the overall population or only from the biomarker-positive population even during the interim decision period conferred a major advantage, since the total trial period did not differ substantially from that of trials employing the non-enriched design. By contrast, the non-enriched design should be used in settings in which the follow-up period is expected to be longer than the recruitment period, since the total trial period was notably shorter than that of the adaptive enrichment design. Furthermore, the utmost care is needed when the distribution of patient recruitment is concave, i.e., when patient recruitment is slow during the early period, since the total trial period is extended.

**Conclusions:**

Adaptive enrichment designs that entail continued recruitment methods are beneficial owing to the shorter total trial period than expected in settings in which the recruitment period is expected to be longer than the follow-up period and the biomarker-positive population is promising.

## Background

The development of targeted therapies is highly attractive to numerous investigators in pharmaceutical research owing to the rapid advancement of personalized medicine [1]. In the clinical phase, biomarker information based on gene expression contributes to identification of subgroups. Generally, gene expression profiles are analyzed by integrated omics technologies [2]. In confirmatory clinical trials, there are numerous examples of therapies that showed a benefit for only a specified subgroup. For instance, gefitinib significantly improved patients with advanced pulmonary adenocarcinoma, particularly those in the subgroup that consists of biomarker-positive patients for epidermal growth factor receptor (EGFR) inhibitor with pulmonary adenocarcinoma, whereas no improvement was observed in patients who tested negative [3]. This trial was conducted for both the biomarker-positive and -negative patients, although this was not pre-specified to demonstrate the benefit for only the biomarker-positive patients. In contrast, as another example, crizotinib significantly improved patients in the subgroup under a clinical trial in which only biomarker-positive patients with advanced anaplastic lymphoma kinase (ALK)-positive nonsquamous non-small cell lung cancer (NSCLC) were set as the targeted population [4]. In order to develop targeted therapies, a complex clinical trial design considering the existence of a promising biomarker to formulate the subgroup is required. Like in the gefitinib example [3], we aimed to set up a clinical trial targeting both the biomarker-positive and -negative patients.

Adaptive enrichment designs could be useful for the development of targeted therapies when the overall population (*O*), which consists of both biomarker-positive (*P*) and biomarker-negative (*N*) populations, is set as the target and particularly when *P* is promising, but not certain, based on the early phase clinical trial results. These designs provide an opportunity to stop the recruitment process for patients in *N* in mid-course and continue enrolling patients in only *P* on the basis of an interim analysis, whereas the classical group sequential design solely provides the opportunity to stop the trial early for efficacy or futility reasons. Accordingly, it is possible to curtail the duration in which patients are exposed to a fruitless treatment if an experimental treatment is overwhelmingly worse than the standard treatment in *N*, but is a benefit in *P*.

We consider an adaptive enrichment design where the endpoint is time-to-event. In this context, there are various advantages with respect to stopping the investigation for *N* in mid-course depending on the recruitment situation, i.e., whether the interim analysis is performed after accumulating the targeted number of patients or not. The advantages can be illustrated with two phase III clinical trial examples from CLEOPATRA and REGATTA studies [5–8]. First, pertuzumab was evaluated in patients with anti-human epidermal growth factor receptor 2 (HER2)-positive metastatic breast cancer in a CLEOPATRA study that incorporated interim analyses [5–7]. The first interim analysis showed a significantly improved progression-free survival [5], and the second interim analysis showed a significantly improved overall survival as well [6]. With respect to patient recruitment, the first interim analysis included 808 patients, satisfying the targeted sample size (TSS) of 800 patients. Thus, the goal of interim analyses was not to decrease the number of recruited patients, which already exceeded the TSS at the time of the first interim analysis. If the adaptive enrichment design is applied in this case, the only advantage is the potential for a reduced follow-up period for patients in *N*.

In contrast, in the REGATTA study, an interim analysis was conducted to investigate the efficacy of the addition of gastrectomy to chemotherapy in advanced gastric cancer patients with a single non-curable factor when the targeted number of patients was not yet reached [8]. The required sample size in the REGATTA study was set to 330 and there was an interim futility criterion, i.e., a hazard ratio of greater than 1.00. Consequently, this trial was terminated owing to futility at the interim analysis based on a hazard ratio (95% confidence interval) for overall survival of 1.09 (0.78 to 1.52). By stopping the trial early, the total number of patients was reduced by approximately half, since the interim analysis included 175 patients. Accordingly, in this case, the adaptive enrichment design is advantageous with respect to decreasing the number of recruited patients as well as curtailing the follow-up period for *N*. However, patient recruitment is halted until the interim decision is made. This increases the duration of the trial and delays the submission of experimental treatments for market approval. In practice, a longer trial duration is a particularly serious drawback of the adaptive enrichment design for pharmaceutical companies.

In this paper, we explore patient recruitment methods for the development of targeted therapies with an adaptive enrichment design. In the setting of the adaptive enrichment design with time-to-event endpoints, data are handled depending on the stage in which each patient is recruited. For example, a patient recruited in the first stage and observed as an event in the second stage will be handled as first-stage data. We consider patient recruitment in the context of a two-stage adaptive enrichment design. To the best of our knowledge, patient recruitment within the adaptive enrichment design setting typically needs to be halted pending an interim decision, and is resumed after the interim decision has been made. Here, we focus on patient recruitment that is continued for patients in *O*, or only for patients in *P*, even during the period pending interim decisions after the first stage. We performed a simulation study to demonstrate the major drawback of using the typical recruitment method with an adaptive enrichment design as well as the advantages of incorporating continued recruitment methods into this design.

## Methods

### Review of the adaptive enrichment design with time-to-event endpoints

A number of adaptive designs in confirmatory clinical trials have been investigated, such as the adaptive treatment selection design, adaptive sample size modification design, and adaptive enrichment design [9]. In this paper, we consider the adaptive enrichment design for the development of targeted therapies. In this design, an interim analysis is conducted to identify whether *O* or only *P* should be included in the subsequent stage. For time-to-event endpoints, constructing test statistics for the final analysis by handling data depending on the stage at which each patient is recruited is needed to control the type I error rate [10]. Let $p^{\{g\}}_{k}$ denote the p-value for stage *k* in population *g*∈{*O,P*}. The final analysis with a one-sided significance level of *α* under a null hypothesis $H^{\{g\}}_{0}$ will be conducted following an inverse combination method [11] as follows:

1 $$\begin{array}{@{}rcl@{}} C\left(p_{1}^{\{g\}}, p_{2}^{\{g\}}\right) &=& w_{1}\Phi^{-1}\left(1-p_{1}^{\{g\}}\right)\\&+&w_{2}\Phi^{-1}\left(1-p_{2}^{\{g\}}\right) > \Phi^{-1}(1 - \alpha) \,  \end{array} $$

where *Φ*(·) denotes the cumulative distribution function of the standard normal distribution and *w*_*k*_ denotes the pre-specified weight for each stage, defined as $w_{k}=\sqrt {\mathstrut I_{O, k}/{\sum \nolimits }_{j} I_{O, j}}$ with information level *I*_*O,k*_ at stage *k* in the calculation of $C\left (p_{1}^{\{O\}}, p_{2}^{\{O\}}\right)$ under the assumption that the p-values from each stage are independent. Note that *I*_*O,k*_ is set as the pre-specified number of events that corresponds to information from *O* in the context of time-to-event endpoints. Here, *w*_*k*_ is also used in the calculation of $C\left (p_{1}^{\{P\}}, p_{2}^{\{P\}}\right)$ under the assumption that the pre-specified number of events for *P* is the same as *I*_*O,k*_. The Hochberg [12] procedure is applied to control the familywise type I error rate at the final analysis. Let $p_{k}^{\{O, P\}}=\min \left [2\cdot \min \left (p_{k}^{\{O\}},p_{k}^{\{P\}}\right),\max \left (p_{k}^{\{O\}},p_{k}^{\{P\}}\right)\right ]$ denote the p-value for the intersection hypothesis $H_{0}^{\{O, P\}}=H_{0}^{\{O\}} \cap H_{0}^{\{P\}}$. Following the closure principle [13], $H_{0}^{\{O\}}$ is rejected if $C\left (p_{1}^{\{O, P\}}, p_{2}^{\{O, P\}}\right) < \alpha $ and $C\left (p_{1}^{\{O\}}, p_{2}^{\{O\}}\right) < \alpha $ are satisfied and $H_{0}^{\{P\}}$ is rejected if $C\left (p_{1}^{\{O, P\}}, p_{2}^{\{O, P\}}\right) < \alpha $ and $C\left (p_{1}^{\{P\}}, p_{2}^{\{P\}}\right) < \alpha $ are satisfied in cases where *O* is determined to be continued, whereas $H_{0}^{\{P\}}$ is rejected if $C\left (p_{1}^{\{O, P\}}, p_{2}^{\{P\}}\right) < \alpha $ and $C\left (p_{1}^{\{P\}}, p_{2}^{\{P\}}\right) < \alpha $ are satisfied in case where only *P* is determined to be continued and *N* is terminated at the interim analysis.

In contrast, typical hazard ratios are often used to conduct interim analyses for the sake of simplicity, although several approaches have been developed to make interim decisions. In this paper, we consider the interim decision rule using the hazard ratios $HR^{\{g\}}_{\text {int}}$ with the thresholds *η*^{*g*}^ for population *g*∈{*O,P*} at the interim as follows: (i) stop the trial for futility if *HR*^{*O*}^_int_>*η*^{*O*}^ and *HR*^{*P*}^_int_>*η*^{*P*}^, (ii) continue *O* if *HR*^{*O*}^_int_<*η*^{*O*}^ regardless of the value of $HR^{\{P\}}_{\text {int}}$, (iii) continue only *P* if *HR*^{*O*}^_int_>*η*^{*O*}^ and *HR*^{*P*}^_int_<*η*^{*P*}^, where the experimental treatment is beneficial against the control if the hazard ratio is less than 1.00.

### Patient recruitment strategies in the adaptive enrichment design

In the context of time-to-event endpoints, it is necessary to consider the recruitment and follow-up periods to accumulate the targeted number of events. To establish a randomized parallel-group clinical trial with equal allocation comprising the experimental (*E*) and control (*C*) groups, it is widely popular to estimate the TSS to achieve 100·(1−*β*)*%* power under a one-sided test with a significance level of *α* according to the Schoenfeld [14] formula as follows:

$$\begin{array}{@{}rcl@{}} \text{TSS} = \frac{ 4 \left\{ \Phi^{-1}(1 - \alpha) + \Phi^{-1}(1 - \beta)\right\}^{2} }{ \delta^{2}} \times \frac{2}{ S_{E}(t) + S_{C}(t) }  \end{array} $$

where *δ* is the log-transformed hazard ratio and *S*_*E*_(*t*) and *S*_*C*_(*t*) are the survival probabilities for *E* and *C* groups at time *t*. In this formula, the TSS is estimated by considering the expected event rates. The TSS varies depending on the recruitment and follow-up periods during the estimation of the expected event rates, in contrast with the TSS calculation in the setting in which the number of events is considered a binary endpoint.

**Fig. 1:**
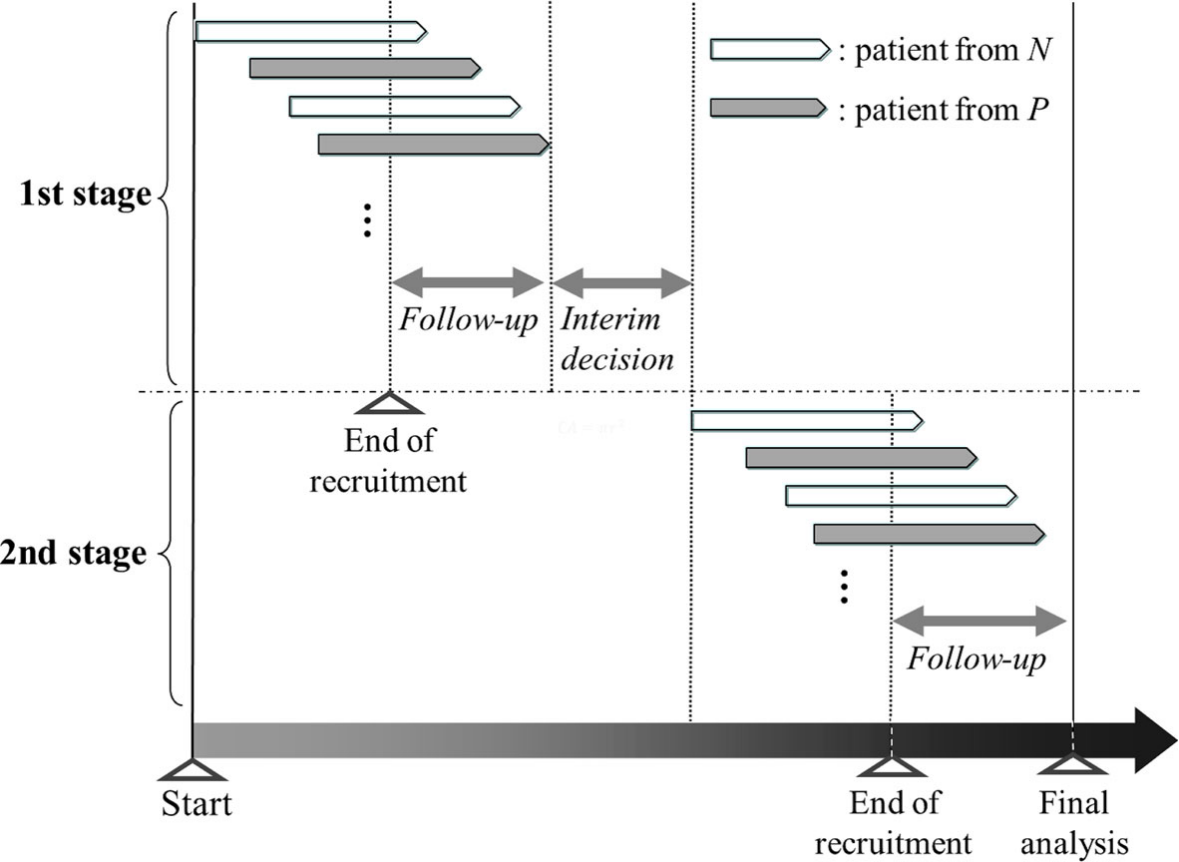
Typical patient recruitment for the adaptive enrichment design. *P*, biomarker-positive population; *N*, biomarker-negative population

**Fig. 2:**
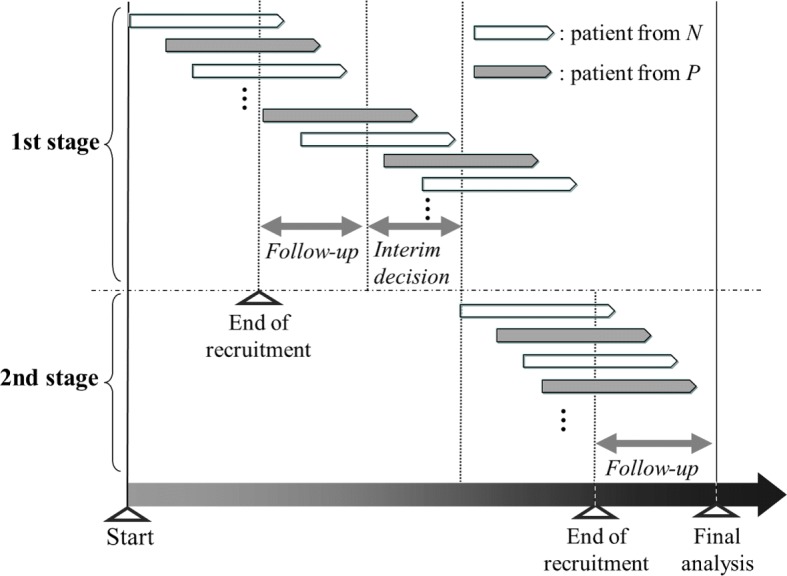
Continued patient recruitment to recruit patients from *O* for the adaptive enrichment design. *P*, biomarker-positive population; *N*, biomarker-negative population

**Fig. 3:**
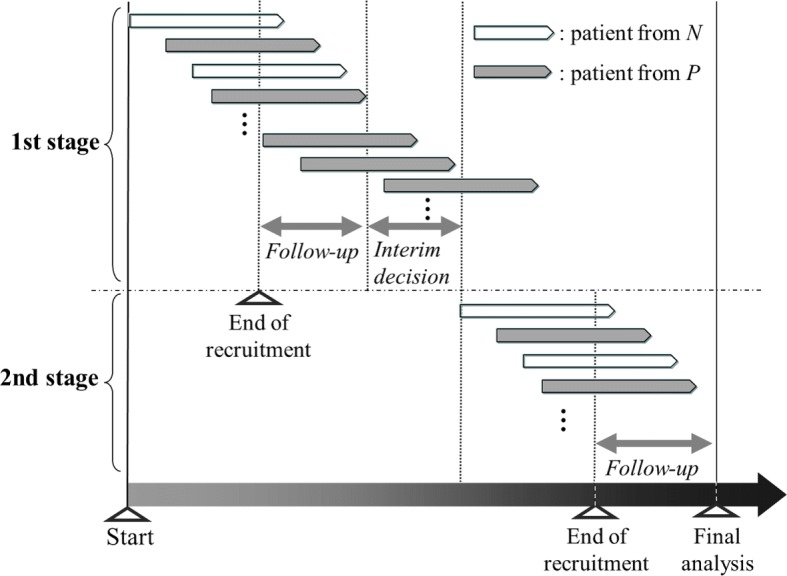
Continued patient recruitment to recruit patients from *P* for the adaptive enrichment design. *P*, biomarker-positive population; *N*, biomarker-negative population

As described in the Background, whether the required number of patients has been recruited before the interim analysis is the key issue in the adaptive enrichment design. In this paper, we consider a method in which patient recruitment is halted until the interim decision regarding the continuation of the population is made, as supposed in most methodology papers considering adaptive enrichment designs, e.g., Jenkins et al. [10], Brannath et al. [15], Friede et al. [16], Krisam and Kieser [17], and Uozumi and Hamada [18]. In contrast to cases where the required number of patients has been recruited before the interim analysis, such as in the CLEOPATRA study [5], this is expected to result in longer total trial periods owing to the pause in recruitment to make the interim decision. As illustrated in Fig. 1, the typical recruitment method in the adaptive enrichment design has to be halted from the end of recruitment at the first stage until recruitment is resumed at the beginning of the second stage. In contrast, the alternative recruitment methods enable continued recruitment, even during the period from the end of recruitment at the first stage to the interim decision based on the interim analysis results, as illustrated in Figs. 2 and 3. The first method shown in Fig. 2 allows continued recruitment for patients in *O* during the interim decision period. In the second method described in Fig. 3, recruitment is continued for patients in *P* with the belief that *P* is promising whereas *N* is harmful. A benefit of the continued recruitment method for *P* is that it provides the opportunity for investigators to stop *N* early owing to futility, under the assumption that the experimental treatment in *P* is relatively promising. Stopping *N* early reduces the actual number of patients recruited and curtails the follow-up period for biomarker-negative patients enrolled before the interim analysis. Note that no modification to the statistical methods used for the adaptive enrichment design is required when the continued recruitment methods illustrated in Figs. 2 and 3 are applied.

## Results

### Simulation study

We conducted simulations to investigate the operating characteristics of three types of patient recruitment strategy using an adaptive enrichment design with respect to the expected numbers of patients and total trial periods. The two alternative methods that allow continued recruitment for *O* or only *P* are assessed. In our simulations, we consider a randomized, parallel-group clinical trial with two arms, comprising *E* and *C* groups.

#### Simulation setting

With respect to the population, *O* includes 50% of *P* in a setting wherein a promising biomarker exists and in which the targeted therapy is beneficial only for *P*. We set the survival probability in *C* at the end of follow-up to 20%. The survival probability in *E* at the end of follow-up was derived on the basis of the assumed hazard ratio between *E* and *C*. With respect to the assumed hazard ratios, we consider five scenarios for applications to the development of targeted therapies. The assumed hazard ratios for *P* are set to 0.50 across every scenario, whereas those for *N* are set to (Scenario 1) 0.50, (Scenario 2) 0.90, (Scenario 3) 1.00, (Scenario 4) 1.11, and (Scenario 5) 1.43. We set the sample size for *O* to 330 following a previous example [8]. When the pre-specified number of events set to 270 occurs, the final analysis is conducted with a one-sided significance level of 2.5% following the closure principle [13] and using the Hochberg [12] procedure with respect to the multiplicity between *O* and *P*. An interim analysis is performed after half of the pre-specified events occur in order to determine whether subsequent recruitment includes *O* or is restricted to *P*. We do not consider early termination if *E* is deemed promising for efficacy. With respect to the thresholds in the interim decision rule, *η*^{*O*}^ and *η*^{*P*}^ are each set to 1.00 for the sake of simplicity.

Durations of patient recruitment *R* and follow-up *F* are set to (*R,F*)=(2,4),(4,2),(6,2), respectively. We consider both uniform and non-uniform recruitment patterns over the interval 0 to *R*. When it comes to the non-uniform recruitment pattern, the truncated exponential distribution is assumed with the cumulative distribution function as follows:

$$\begin{array}{@{}rcl@{}} F(t) = \frac{ 1 - \text{exp} (-\gamma t) }{ 1 - \text{exp} (-\gamma R)},  \end{array} $$

where *γ* denotes the shape parameter, with *γ*≠0 [19]. The distribution of recruitment is convex if *γ*>0 and concave if *γ*<0. For instance, for *γ*<0, the recruitment speed is slow at the early period. In this simulation setting, the slow recruitment pattern, with *γ*<0 and using the continued recruitment methods, also shows that recruitment accelerates during the interim decision. In contrast, the fast recruitment pattern, with *γ*>0 and using the continued recruitment methods, implies that recruitment slows down during the interim decision period. For convex and concave recruitment patterns, we set *γ* to 2 and − 2. Furthermore, we consider the duration for data lock between stages. The duration from the data lock of the first stage to the beginning of the second stage, i.e., interim decision period, is set to 0.2.

The timing of the interim analysis is mainly assumed as 50% of the pre-specified events based on the example [8]. We further evaluate the trade-off between timing of the interim analysis and the accuracy of the interim results. The timing of the interim analysis is set to 25%, 50%, and 75% of the pre-specified events. The accuracy of the interim hazard ratio for *P* is assessed.

#### Simulation results

We evaluated the operating characteristics of the two recruitment methods in the adaptive enrichment designs in terms of the expected numbers of patients and expected total trial periods. The characteristics were quantified based on 10,000 simulations.

**Fig. 4:**
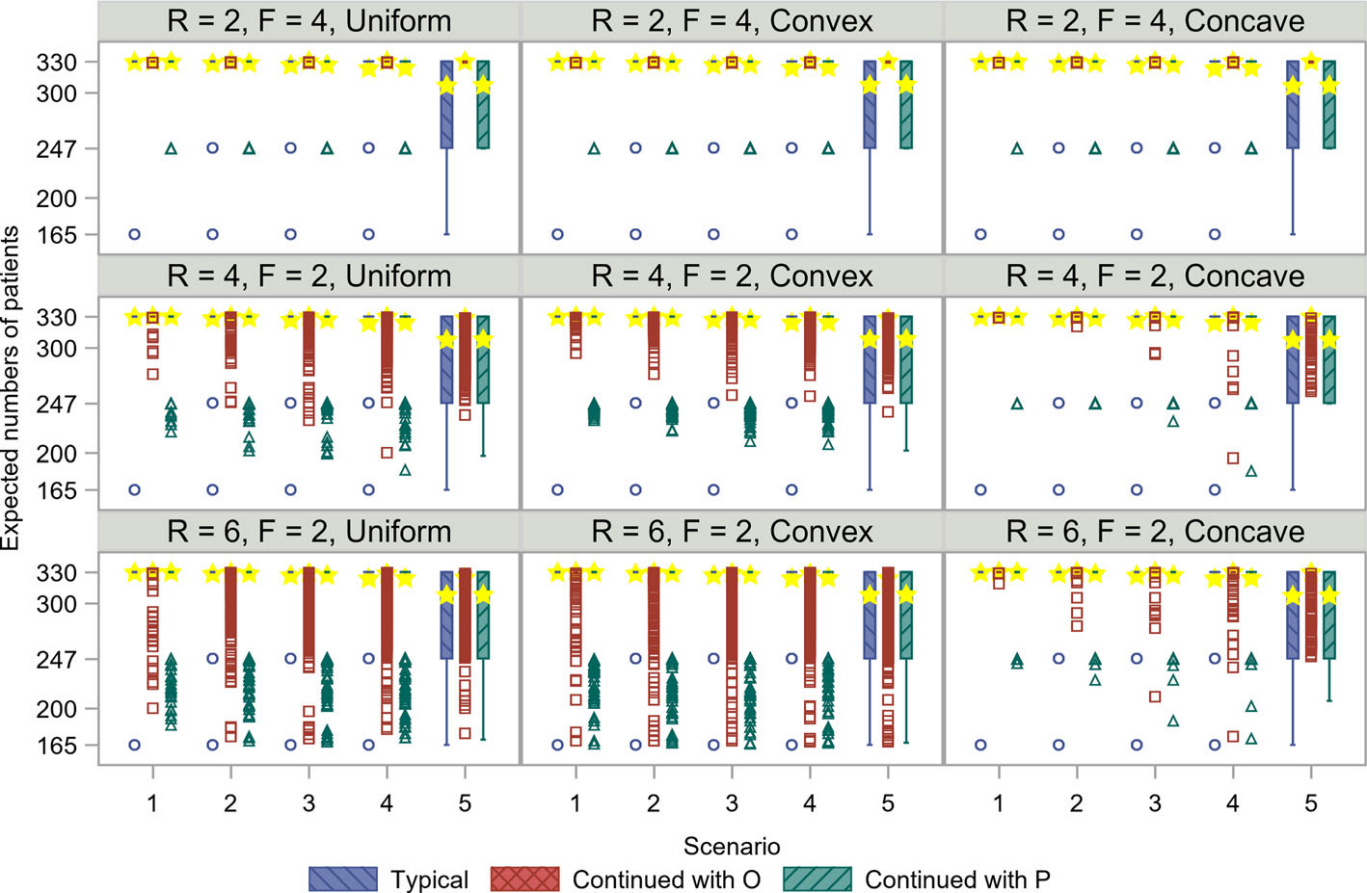
Comparison of expected numbers of patients in *O* for various recruitment strategies. Boxplots with circles (blue), squares (red), and triangles (green) show the results using each recruitment method. Stars (yellow) represent means. The expected number of patients under the non-enriched design was set to 330. *R*, recruitment period; *F*, follow-up period; *O*, overall population; *P*, biomarker-positive population

**Fig. 5:**
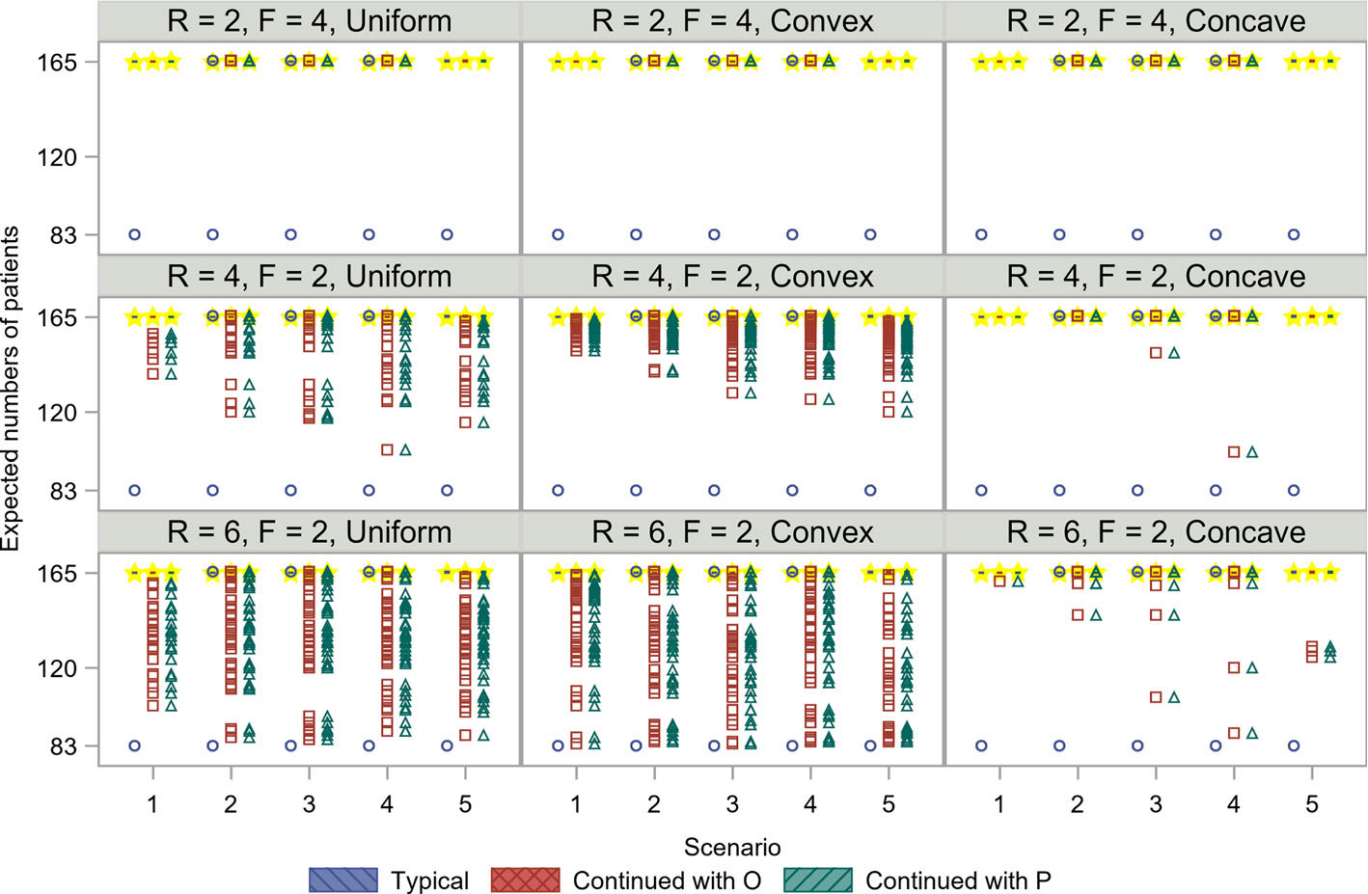
Comparison of expected numbers of patients in *P* for various recruitment strategies. Boxplots with circles (blue), squares (red), and triangles (green) show the results using each recruitment method. Stars (yellow) represent means. The expected number of patients in *O* under the non-enriched design was set to 330. The expected number of patients in *P* was 165 since the prevalence of *P* among *O* was set to 50%. *R*, recruitment period; *F*, follow-up period; *O*, overall population; *P*, biomarker-positive population

Figure 4 shows the expected numbers of patients from *O* using each recruitment method within the adaptive enrichment design assuming uniform, convex, and concave recruitments. In general, the expected number of patients was less than the reference number of 330 when the non-enriched design was employed. The non-enriched design corresponds to the group sequential design that includes early termination only for futility. As shown in Fig. 4, the minimum number of patients is 165 under typical recruitment owing to the possibility of no extra recruitment after recruiting half of the targeted number of patients. The minimum number of patients using continued recruitment with *P* in the setting of *F*>*R* is calculated as 247 which consists of half number of patients before the interim analysis and patients in *P* after the interim analysis. In particular, the number of patients was decreased in Scenario 5, in which the *E* is notably harmful in *N*, as adaptive enrichment for *P* is expected. In the continued recruitment methods, patients are continually enrolled, even during the period in which the interim decision is made, as described in Figs. 2 and 3. The expected number of patients is similar among the recruitment methods in Scenarios 1 to 4, regardless of recruitment patterns, when *F* is expected to be longer than *R*. In Scenario 5, under the setting of *F*>*R*, the expected number of patients using the continued recruitment method to recruit patients from *O* is largest among the three methods. However, the difference is apparent when *R* is expected to be longer than *F*. The number of patients using the continued recruitment methods is expected to be lower, especially when (*R,F*)=(6,2). In particular, the advantage of the continued recruitment methods is observed in Scenario 5 in terms of the similar distributions to the typical recruitment method. The distribution obtained using the continued recruitment method to recruit patients from *P* is more similar to that obtained using the typical recruitment method, than it is to that obtained using the continued recruitment method to recruit patients from *O*. Both continued recruitment methods require more patients than the typical recruitment method owing to the continued recruitment during the interim decision period. The continued recruitment method to recruit patients from *O* particularly needs far more patients than the continued recruitment method to recruit patients from *P* since the continued recruitment is not limited to patients from *P*. Moreover, the difference in the minimum number of patients was notable, even in Scenarios 1 to 4. Figure 4 shows the differences in expected number of patients for the various recruitment patterns. Although the expected and maximum number of patients are similar regardless of the recruitment patterns, the minimum number of patients with the concave recruitment pattern, i.e., *γ*<0, is higher than with the uniform recruitment pattern. On the other hand, the minimum number of patients with the convex recruitment pattern, i.e., *γ*>0, is closer to that with the uniform recruitment pattern. Next, Fig. 5 shows the expected numbers of patients from *P* using each recruitment method. The expected number of patients in *P* is half of 330 since the prevalence of *P* among *O* is set to 50%. Owing to the possibility of early termination for futility after recruiting half of the targeted number of patients, the numbers of patients in *P* using the continued recruitment methods with *O* or *P* were distributed from 83 to 165, especially in the setting of *R*>*F*.

**Fig. 6:**
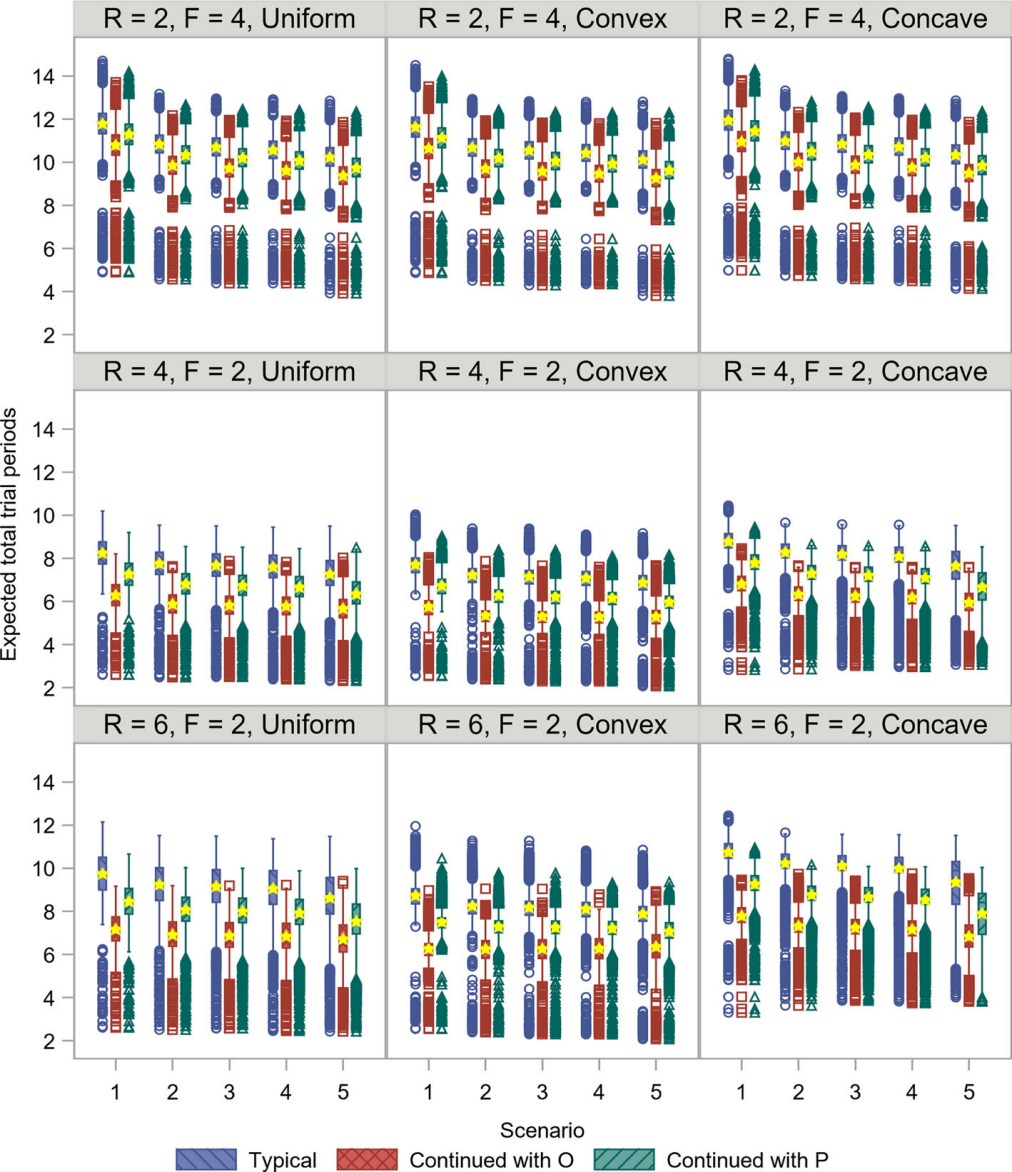
Comparison of expected total trial periods among patient recruitment strategies. Boxplots with circles (blue), squares (red), and triangles (green) show the results using each recruitment method. Stars (yellow) represent means. *R*, recruitment period; *F*, follow-up period; *O*, overall population; *P*, biomarker-positive population

Figure 6 summarizes the expected total trial periods for each recruitment method. Uniform and truncated exponential distributions were assumed to investigate the performance of the recruitment methods. As a reference, the expected total trial period is *R*+*F* when the non-enriched design is employed. In comparison, the expected total trial period was shorter in Scenario 5, since the probability of selecting only *P* is highest among the five scenarios. With respect to the recruitment and follow-up periods (*R,F*), the non-enriched design should be used when *F* is expected to be longer than *R*, since the total trial period using the adaptive enrichment design is much longer than that using the non-enriched design. By contrast, the adaptive enrichment design is applicable when *R* is expected to be longer than *F*, since the total trial period using the adaptive enrichment design does not substantially differ from that of the non-enriched design. These results are particularly apparent when (*R,F*)=(6,2), i.e., when *R* is expected to be much longer than *F*. Despite the similar expected numbers of patients for all of the recruitment methods, the expected total trial period is notably shorter for the continued recruitment methods than for the typical recruitment method; hence, the application of the adaptive enrichment design is attractive when using the continued recruitment methods. In particular, the expected total trial period is shorter using the continued recruitment method with *O*, despite more patients being required. Note, however, that the minimum number of patients is larger when the adaptive enrichment design is applied. In addition, situations in which patient recruitment pattern is concave during *R* should be handled with the utmost care, since a longer total trial period is expected to achieve the pre-specified number of events than the setting in which recruitment follows a uniform or convex pattern.

**Fig. 7:**
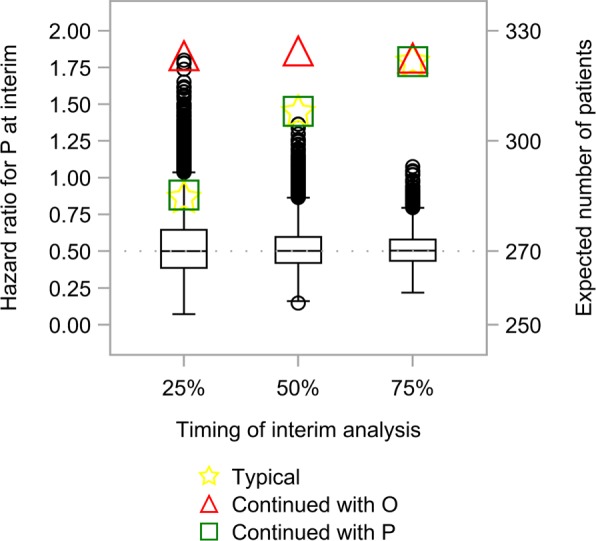
Comparison between timing of the interim analysis and the accuracy of interim hazard ratio for *P*. Boxplots with circles (black) show trade-off between timing of the interim analysis and the accuracy of interim hazard ratio for *P*. Stars (yellow), triangles (red), and squares (green) represent the expected number of patients using patient recruitment strategies. *R*, recruitment period; *F*, follow-up period; *O*, overall population; *P*, biomarker-positive population

Finally, Fig. 7 illustrates the boxplots assessing trade-off between timing of the interim analysis and the accuracy of interim hazard ratio for *P* while providing the expected number of patients assuming uniform, convex, and concave recruitment patterns under the setting of (*R,F*)=(6,2) and Scenario 5. Early interim analysis with enrichment obviously leads to a beneficial result in terms of smaller number of patients required; however, the accuracy is severely decreased owing to the lack of information. This implies that the probability of selecting an inappropriate population in mid-course is increased with early interim analysis. In contrast, accuracy is improved when a late interim decision is made, but the benefit of adaptive enrichment designs is reduced owing to a large number of patients required.

## Discussion

Enrichment designs are generally applied without stopping recruitment; however, we focus on adaptive enrichment designs with emphasis on whether patient recruitment is halted. In this paper, we assessed patient recruitment methods in the context of adaptive enrichment designs for the development of targeted therapies in the setting in which recruitment is halted until an interim decision regarding the continuation of *O* or *P* is made. The adaptive enrichment design with the typical recruitment method results in a longer total trial period than that of the non-enriched design. As an alternative, the continued recruitment methods resolve this issue, i.e., they enable a shorter total trial period. Despite this benefit, the expected number of patients required does not differ between the three recruitment methods. In the continued recruitment methods, recruitment is continued even after the first stage of recruitment. In particular, the continued recruitment method to recruit patients from *O* enables the total trial period to be shortened. However, this method has the corresponding possibility of increasing the anticipated minimum number of patients required. On the other hand, the continued recruitment method to recruit patients only from *P* requires the assumption that *P* is promising; however, it is more attractive in terms of the lower anticipated minimum number of patients. Using the continued recruitment method to recruit patients from *P* is preferred if reducing sample size is more desirable than reducing the trial period. This approach resulted in a similar number of patients to the typical recruitment method. The number of patients differed from that obtained using the continued recruitment to recruit from *O*, particularly under the setting in which *R* is expected to be longer than *F*. With emphasis on total trial period, it is preferable to use the continued recruitment method to recruit patients from *O* should be employed, taking into account the trade-off between sample size and total trial period.

In our simulation study, we considered that the assumed hazard ratios are consistent between stages. According to the draft guidance by the Food and Drug Administration [20], adaptive designs can lead to potential operational biases that result in inconsistency between stages. If potential operational bias is introduced after the interim analysis, we might face a situation in which *N* is stopped at the interim analysis, but in which both *P* and *N* are found to be promising after the interim decision. To ensure trial integrity, access to interim analysis results should be limited to the data monitoring committee members who are independent of the investigators being involved in the trial. The potential operational bias introduced in this scenario will be assessed in future work.

For the sake of simplicity, we did not consider early termination if the experimental treatment was deemed promising with respect to efficacy. In the context of adaptive enrichment designs, there are several types of assumptions for clinical trial phases, such as phase II/III or phase III settings. When early termination for each population is considered in a phase III clinical trial, the O’Brien and Fleming [21] stopping boundary at the time of the interim analysis is calculated with the use of a Lan and DeMets [22] alpha-spending function on the basis of the number of events observed at the data cutoff date. Moreover, we set the proportion of *P* among *O* to 50% for simplicity. The continued recruitment methods reduce the length of the trial period when this proportion is higher than 50%. By contrast, the benefit of these methods is reduced when the proportion is less than 50%. In practice, the thresholds for the hazard ratios or other measures for each population in the interim decision should be carefully set based on thorough simulations for various scenarios, as Gallo et al. [23] pointed out when using simple group sequential designs.

Furthermore, the timing of the interim analysis in the adaptive enrichment design should be considered, similar to the context of a group sequential design [24, 25], because early decisions have the potential risk for over-estimation of treatment effect [26, 27]. Our simulation results indicate that early interim analysis spoils the accuracy of interim results that leads to an incorrect decision. To supplement immature information in mid-course, the potential use of biomarkers or other endpoints associated with the primary endpoint of interest would be useful to drive the adaptive enrichment decision. When overall survival is assumed as a primary endpoint, other time-to-event endpoints such as progression-free survival would be beneficial to strengthen the information level in mid-course in the situation in which progression-free survival is highly correlated with overall survival, as illustrated in Uozumi and Hamada [18]. Although we assumed performing the interim decision with a simple rule, more investigation for the optimal timing should be required when applying the adaptive enrichment design in practice since performance greatly depends on the interim decision rule as shown in Benner and Kieser [28].

Note that the adaptive enrichment design is not encouraged for the development of targeted therapies in some conditions in which the non-enriched design is more appropriate. For instance, the adaptive enrichment design with more than two stages would be much more inefficient in terms of halting the recruitment several times to make interim decisions. Similarly, this is true even using the adaptive treatment selection designs when multiple experimental treatment groups are set as a randomized parallel-group clinical trial.

Our simulation study showed that the adaptive enrichment design is beneficial when *R* is expected to be longer than *F*, while the non-enriched design is recommended when *F* is expected to be longer than *R*. For instance, the adaptive enrichment design is applicable in the setting in which progression-free survival, rather than overall survival, is set as a primary endpoint. Furthermore, the total trial period is particularly long when the distribution of recruitment is concave. In practice, the interim analysis is performed when a pre-specified number of events occurs, regardless of the recruitment pattern, and prolonged recruitment and follow-up periods are considered until the pre-specified number of events is obtained when the recruitment speed is slow. Hence, the application of the adaptive enrichment design is not encouraged when the interim analysis hinders recruitment until the interim decision is made in mid-course, regardless of whether the trial periods is prolonged.

## Conclusions

We evaluated patient recruitment methods in the context of an adaptive enrichment design for developing targeted therapies. The adaptive enrichment design typically requires a longer total trial period than that of the non-enriched design. As an alternative, the continued recruitment methods provide the option to continue recruiting patients from *O* or only from *P* even during the interim decision period when recruitment from *O* is halted. The performance of the continued recruitment methods demonstrates that they may enable adaptive enrichment designs to be applied to a broader range of clinical trials, particularly when *R* is expected to be longer than *F*.

## Data Availability

The datasets used and/or analysed during the current study are available from the corresponding author on request.

## Abbreviations

ALK: Anaplastic lymphoma kinase
*C*: Control
*E*: Experimental
EGFR: Epidermal growth factor receptor
*F*: Follow-up period
HER2: Human epidermal growth factor receptor 2
NSCLC: Non-small cell lung cancer
*N*: biomarker-negative population
*O*: Overall population
*P*: biomarker-positive population
*R*: Recruitment period
TSS: Targeted sample size
